# Characterizing News Report of the Substandard Vaccine Case of Changchun Changsheng in China: A Text Mining Approach

**DOI:** 10.3390/vaccines8040691

**Published:** 2020-11-17

**Authors:** Ping Zhou, Yao He, Chao Lyu, Xiaoguang Yang

**Affiliations:** 1 Department of Hospital Management, Key Laboratory of Health Technology Assessment of National Health Commission (Fudan University), School of Public Health, Fudan University, Shanghai 200032, China; zhouping@fudan.edu.cn (P.Z.); 14301020033@fudan.edu.cn (C.L.); 2 Department of Global Health, School of Public Health, University of Washington, Seattle, WA 98195-7965, USA; yaohe@uw.edu

**Keywords:** substandard vaccine case, news report, text mining, topic model, sentiment analysis

## Abstract

*Background:* The substandard vaccine case of that broke out in July 2018 in China triggered an outburst of news reports both domestically and aboard. Distilling the abundant textual information is helpful for a better understanding of the character during this public event. *Methods*: We collected the texts of 2211 news reports from 83 mainstream media outlets in China between 15 July and 25 August 2018, and used a structural topic model (STM) to identify the major topics and features that emerged. We also used dictionary-based sentiment analysis to uncover the sentiments expressed by the topics as well as their temporal variations. *Results*: The main topics of the news report fell into six major categories, including: (1) Media Investigation, (2) Response from the Top Authority, (3) Government Action, (4) Knowledge Dissemination, (5) Finance Related and (6) Commentary. The topic prevalence shifted during different stages of the events, illustrating the actions by the government. Sentiments generally spanned from negative to positive, but varied according to different topics. *Conclusion*: The characteristics of news reports on vaccines are shaped by various topics at different stages. The inner dynamics of the topic and its alterations are driven by the interaction between social sentiment and governmental intervention.

## 1. Introduction

Vaccination is the most effective medical approach that can be used to eliminate suffering from the health and financial burdens caused by large-scale infectious disease transmission. While vaccination usually accompanies wide societal concern, the mass media also plays an influential role on its acceptance [1,2,3]. The Strategic Advisory Group of Experts on Immunization Working Group on Vaccine Hesitancy listed communication and media environment as a key influence on vaccine hesitancy [4,5]. From a research perspective, news reports are a very important vessel for information, as they convey the knowledge, attitudes, and sentiments of a society [6,7,8,9]. Such an abundance of information has been acknowledged by many researchers in the area of vaccines, and studies have tried to reveal the underlying factors that influence vaccination acceptance based on the trace implications of news texts. For example, Becker et al. measured confidence in vaccinations using a multinational media surveillance system [10], and Faasse et al. analyzed the influence of news coverage and Google searches on Gardasil adverse event reporting [11], among other approaches.

Vaccine incidents or related events are an important aspect of vaccine research, particularly when covered by the media and reflecting extensive concern from the society [12,13,14], as they can have a potential impact on vaccination confidence [15,16]. These events can cause average citizens with little professional knowledge on vaccinations to worry, particularly for their children [17,18,19]. On 15 July 2018, a scandal broke out when the Changchun Changsheng Biotech Co. Ltd., one of the major vaccine manufacturers in China, was confirmed to have fabricated production and inspection records and arbitrarily changed process parameters and equipment during its production of freeze-dried human rabies vaccines [20]. Within a week, a huge wave of concern and discussion was raised from mass media. This event drew the attention of the Chinese President Xi Jinpin, who characterized the scandal as “veiled in nature and shocking” and demanded a thorough investigation on 24 July [21]. In the meantime, the World Health Organization also addressed the importance of vaccinations, and called for actions on regulation [22]. The case reached a conclusion on 16 August when the standing committee of the Communist Party of China, the top power authority of China, held more than 40 government officials accountable, including seven at the provincial or ministry level [23], and the Changsheng Limited was ordered to pay about 9.1 billion yuan (USD 1.3 billion) in penalties on 16 October [24].

As a shocking incident, the case of the substandard vaccine by the Changchun Changsheng Company (referred to as the “Changsheng case” for short) triggered an outburst from news reports both in China and aboard, and the incident provides an opportunity to inspect how the media reacts to a sensational public health event. To investigate the large scale and great diversity of the news reports, we employed computer-assisted text mining tools to review the news texts directly. We believe such innovative method could potentially reduce laborious human review or coding work, and could lead to a better presentation of the characteristics of the reporting on the Changsheng case from a holistic perspective.

Topic is an important entry point to inspecting the characteristics of news reports [25,26,27]. In regards to the Changsheng case, the news reports comprised a great variety of topics such as vaccine safety, weak regulating systems, as well as the financial misconduct of the company. Such topics, characterized by proportion, temporality, and sentiment, are helpful to understanding the inner structure, temporal variation, and sentiment of the reports. Specifically, we want to focus on the following research questions:

RQ1: What topics emerged in the news reports during the Changsheng case, and how were they are featured in terms of prevalence and key words?

RQ2: How did the different topics change over time?

RQ3: What sentiments were expressed through the media and how did they change over time from a topical perspective.

In this paper, we first introduce the methods used for analyzing the news report texts. Then we examine the media presentation of the event, including the major topics that emerged from the news reports and their distribution over time. Sentiments present in the news reports were identified based on keywords from temporal and topical perspectives. Finally, we interpret the research findings and draw conclusions.

## 2. Materials and Methods

Text mining approaches were used in this study to characterize news reports of the Changsheng case. Specifically, we tried retrieving the quantified features, like topic prevalence, temporal alteration and sentiment propensity, and demonstrated the features statistically to provide a full picture of the case. Retrieving information from news texts can be challenging, as it is highly unstructured compared with other semi-structural text sources such as legal documents [28], patent documents [29], or electronic health records [30]. In this case, we used a topic modeling approach to deconstruct the news contents, and a lexicon-based method to analyze the news sentiments. We tried to analyze a broad selection of the mainstream newswires inside China to reduce preference or bias from any individual news source.

### 2.1. Materials

We designated the data collection period between 15 July and 25 August 2018, which covered the break out of the event until the official conclusion. We identified 2211 news articles related to the Changsheng case from 384,254 pieces of news published by 83 media outlets in China during the specified period (see Supplementary Materials for detail). The news texts were processed by conventional natural language processing procedures including word segments, removals of numbers, punctuation, and stop words [31]. The minimum word length was kept to two. The final corpus contained the columns of title, date, source, and content for each included news article.

### 2.2. Topic Model: Primary Analytical Approach

#### 2.2.1. Overview of Topic Model

Topic model analysis is an important approach by which to inspect a large quantity of textual data [32]. It provides an intuitive way of identifying what topics potentially exist in the corpus and captures quantities of interest [33]. Such identification can transfer the unstructured textual data into a low-dimension quantitative feature, and could be combined with other methods such as regression [34], time series [34], and sentiment analysis [35].

**Figure 1:**
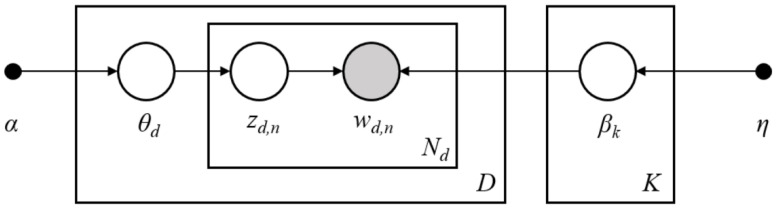
Textual data generative mechanism of the Topic Model [33].

The topic model method assumes that there are a number of potential topics available during the collection of documents, and that each document or word belongs to a certain topic with a different probability [36]. The classical topic model, basing on latent dirichlet allocation (LDA), assumes that specific document is generated by first nominating a topic from the potential topic-document distribution, then selecting a word from the potential word-document distribution, as shown in Figure 1. Assuming there are N documents, K topics, and V words in the whole corpus, θ is the length-K per document-topic distribution for document d, β is the length-V per topic-word distribution for a certain k-th topic, and $Z_{d,n}$ is the selected topic from which the observed words $W_{d,n}$ are chosen [36]. α and *η* are the hyperparameters initially set for model fitting.

The mathematic definition of the classic topic model proposed by Blei et al. is as follow:

θ~Dir(α)

β~Dir(β)

$$Z_{d,n}|\theta_{d}\sim Mutinomial\left(\theta_{d}\right)$$

$$W_{d,n}|Z_{d,n}\sim Multinomial\left(\beta_{Z_{d,n}}\right)$$

Fitting the topic model helps us identify the parameters from a given corpus. For domain-specific research, the most interesting measured quantity is the θ: the proportion of topics relevant to a certain document [33]. Using a statistical aggregation, we can calculate the prevalence of the topics in the whole corpus then probe the semantic structure of the corpus [33].

#### 2.2.2. Structural Topic Model-Based Data Analyses

Topic models are widely applied to a variety of text formats such as newspapers [37,38], patent contents [29], social media [39,40], research articles or reports [41,42], and so on. As an extension of the topic model, the recently developed structural topic model (STM for short) could provide a way of quantifying the effects of document properties (e.g., time of creation, sources) to a specific topic’s prevalence, which is useful for exploring the features of the topics [33,43]. Robert et al., the author of the STM, proposed the model as follows:

$$\theta_{d}|\left(C_{d,\gamma },\Sigma \right)\sim LogisticNorm\left(C_{d,\gamma },\Sigma \right)$$

$$\beta_{d,k}\propto exp\left(m+k_{k}+k_{g,d}+k_{k_{g,d}}\right)$$

$$Z_{d,n}|\theta_{d}\sim Multinomial\left(\theta_{d}\right)$$

$$W_{d,n}|Z_{d,n}\sim Multinomial\left(\beta_{Z_{d,n}}\right)$$

The format and data generation mechanisms are similar between the LDA and STM. The major difference is the change in topic prevalence from Dirichlet distribution into logistic normal distribution, which can incorporate covariates. The parameters $k_{k},\;k_{g,d},\;k_{k_{g,d}}$ represent the specific deviations of the topics, covariates, and interaction topic-covariates, respectively.

In this paper, we used the STM to investigate: (1) what topics emerged from the news reports that were related to the Changsheng case, (2) the quantitative prevalence of the topics from all the reports, and (3) how topic prevalence changed over time. Selecting the suitable number of topics (K) is a challenge in topic model analysis. Despite this, there are quantitative criteria to support selection [32,37], and most studies eventually rely on human judgement [34,37,41]. In this study, we followed the method of Roberts et al. [33], using the build Semantic Coherence and Exclusivity to get an overview of the coverage of topics under different K, then determined the final topic number by manually reviewing the results.

#### 2.2.3. Sentiment Analysis

Sentiment polarity and strength [44] on the news reports of the Changsheng case were calculated from both time and topical perspectives to describe the overall sentiment expression and its variation across the whole event. We also identified the top sentiment terms that contributed to the news text [45]. In this paper, we employed a lexicon-based analysis [46] to calculate the quantitative sentiment propensity of the news report. The lexical dictionary created by the Dalian University of Science and Technology (DUST) [47] was used in this study with minor adaptations. For example, we removed the word “Changsheng” (which means “long life” in Chinese), a very positive word in the DUST dictionary, while incorporating new terms that appeared in the reporting on Changsheng case such as “violated the moral bottom line” (mentioned by Premier Li Keqiang) as a passive word [47].

The above works were implemented using the R (3.4.4) programming language and packages. Specifically, the fitting and visualization of the topic model were conducted using the *stm* [48] and *stminsights* [49] packages, while the sentiment analysis was implemented using the *tidytext* package [50].

## 3. Results

### 3.1. News Occurrence of the Case

**Figure 2:**
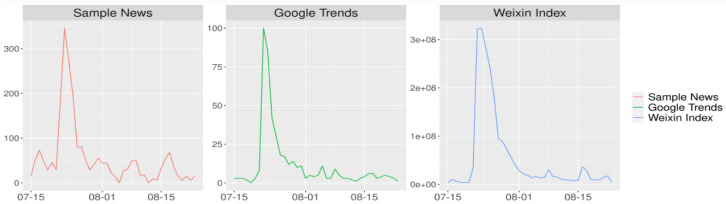
Temporal trends of the news reports included in the study with the corresponding Google trends and Weixin index, indicating the social attention to this incident.

The first chart in Figure 2 shows the change in the amount of news reporting over the time period that the study sample covered (15 July to 25 August 2018) in the Chinese context. Compared to Google Trends, which shows the popularity of topics on the internet (second chart), and the Weixin Index (the most popular social media mobile application similar to WhatsApp in China), which shows the popularity of items on the mobile internet (third chart), the time distribution of the news reports included in this study matched that of the internet and mobile internet closely.

### 3.2. Topics that Emerged from the Corpus

#### 3.2.1. Overall Topical Presentation

Table 1 shows the 17 topics that automatically emerged from fitting the STM, as well as their proportion in the corpus. We listed the top 10 words (ranked by β_k in Figure 1) belonging to each topic, added a representative label to each topic, and categorized them into six groups.

**Figure 3:**
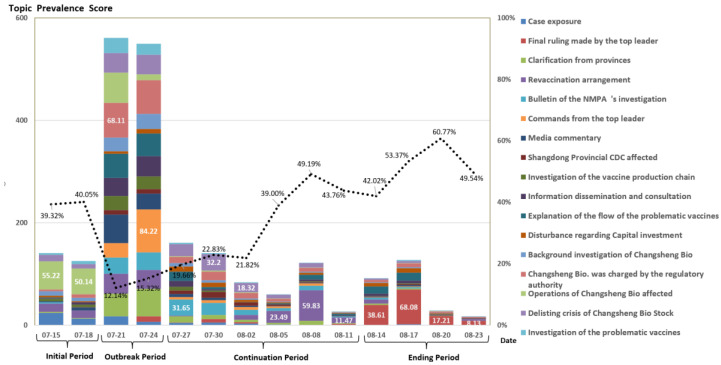
Prevalence and temporal variation of the topics. Note: The dotted line is the proportion of the highest prevalence topics at a certain time point.

To discover how the topics of news reporting changed over time, we divided the observation period into three-day intervals to calculate the proportional distribution of the 17 reporting topics within each interval. Based on the topic distribution, we categorized reports into four periods, namely, the initial period, outbreak period, continuation period, and ending period (Figure 3).

During the initial period (15–20 July), most news reports were case investigations, and the topics of more than half the reports regarded Operations of Changsheng Bio affected (Shenzhen Stock Exchange) and Case Exposure (53.51%).

During the outbreak period (21–25 July), the amount of reporting increased rapidly once the president addressed the case, and the distribution of each topic was fairly similar. Some of the major topics were Changsheng Bio Charged in the Investigation (12.07%), Commands from the Top Leader (10.14%), Clarification from Provinces (8.78%), Explanation of the Flow of the Problematic Vaccines (8.24%), and Media Commentary (7.79%). Government entities at all levels and various sectors in society were highly concerned with the Changsheng case during this period. The peak of media attention lasted for five days, then the number of news reports started to decrease on 26 July.

During the continuation period (26 July–12 August) the dominant topics of reporting shifted towards Results of the Investigation of the National Medical Products Administration (NMPA) and Revaccination Arrangement of the National Health Commission (NHC). The number of reports on these two topics grew rapidly and became mainstream. During ending period (after 13 August), the amount of reporting on the Changsheng case experienced little fluctuation, and the major topic remained Final Ruling (42–61%). Once the State Council published the progress of their investigation into the Changsheng case on 6 August, and the amount of reporting decreased rapidly.

#### 3.2.2. Time Trends of the Topic Categories

**Figure 4:**
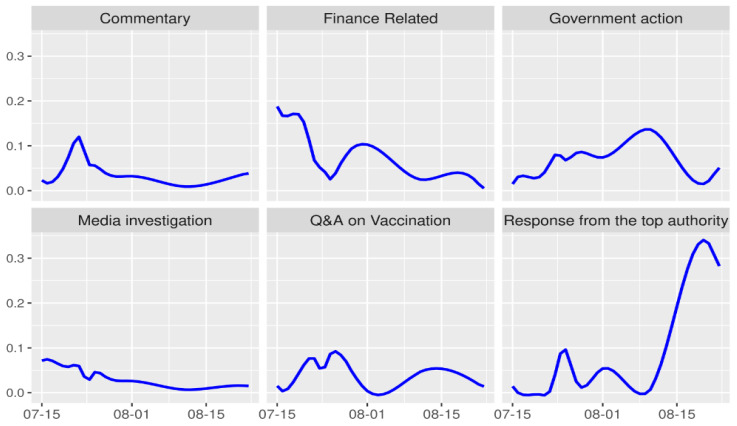
Time trends of the topic categories.

Figure 4 shows the trends of topic categories over time to further illustrate the progress of the case. During the observed period, the prevalence of topics in the Media Investigation category decreased steadily over time. The prevalence of topics in the Commentary category increased rapidly during the initial period, reaching its peak on 22 July (eight days after the case exposure), then decreased rapidly afterwards and remained at a low level towards the end. The prevalence of the Q&A of Vaccination topics fluctuated over time, peaking when the authorities officially announced the investigation results, then decreasing steadily after the second peak. The category of Finance Related received a lot of media attention at the beginning of the initial period, but the prevalence of topics in this category decreased and fluctuated afterwards.

The prevalence of topics in the Response from the Top Authority and Government Action category increased following the initial case exposure. Three peaks in the prevalence of Response from the Top Authority appeared around the 10th, 20th, and 35th days following exposure, which corresponded to the critical time points when the president issued commands, the State Council published the progress of the investigation, and the president announced the final ruling, respectively. The third peak indicates that the final ruling from the president almost dominated news reporting at that time, after which the media attention to this case would start to dissipate. The prevalence of topics in Government Action increased steadily and peaked on the 25th day following exposure.

### 3.3. Sentiment Analysis of the Case

#### 3.3.1. Daily Sentiment Score

**Figure 5:**
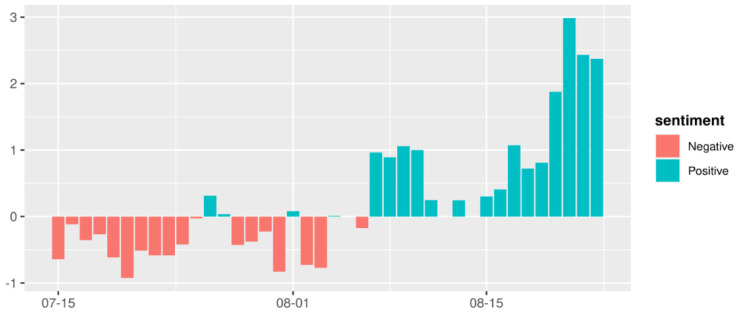
Daily sentiment score of the news.

We found that, despite some fluctuation, sentiment was mostly negative during the first 23 days following the initial case exposure, and that the absolute values of the negative sentiment scores were exceptionally high on the 1st, 6th, 17th, 19th, and 20th days, when there was extensive discussion in the media. Following the 24th day, sentiment became increasingly positive, and there was a sharp increase in positive sentiment towards the end of the observation period (Figure 5).

#### 3.3.2. Sentiment by Topic

**Figure 6:**
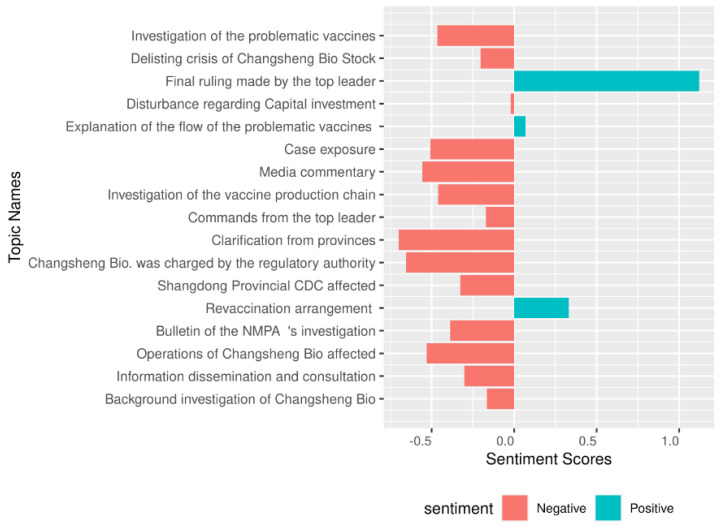
Sentiment scores of the 17 topics.

According to our sentiment analysis by topics (Figure 6), the news reports on Revaccination Arrangement, Explanation of the Flow of the Problematic Vaccines, and Final Ruling Made by the Top Leader expressed positive sentiment, particularly those on Final Ruling. Such topics included the confirmative attitudes of the government, such as “guarantee”, “adamant”, and “crystal clearly”, among others. While news reports on the rest of the topics mostly expressed negative sentiment, particularly on Operations of Changsheng Bio Affected (Shenzhen Stock Exchange), Changsheng Bio was Charged by the Regulatory Authority, Clarification from Provinces, and Media Commentary. These results reflected the strong negative attitudes from the government, society, and media towards the Changsheng case.

#### 3.3.3. Most Frequently Used Sentiment Words

**Figure 7:**
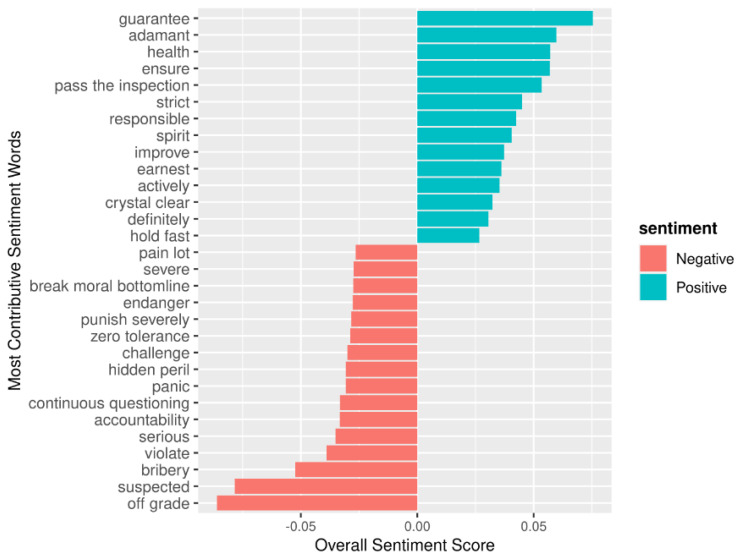
Sentiment words used most frequently in the corpus.

Figure 7 shows the most frequently used sentiment words from the news texts. In terms of negative sentiment, the most frequently used words were “off grade”, “suspected”, “bribery”, and “moral bottom line”, with contributions of −0.086, −0.078, −0.052, and −0.039, respectively. In terms of positive sentiment, the most frequently used words were “guarantee”, “adamant”, “health”, and “ensure”, with contributions of 0.075, 0.059, 0.57, and 0.056, respectively (Figure 7).

#### 3.3.4. Temporal Alteration in the Contribution of the Sentiment Words

**Figure 8:**
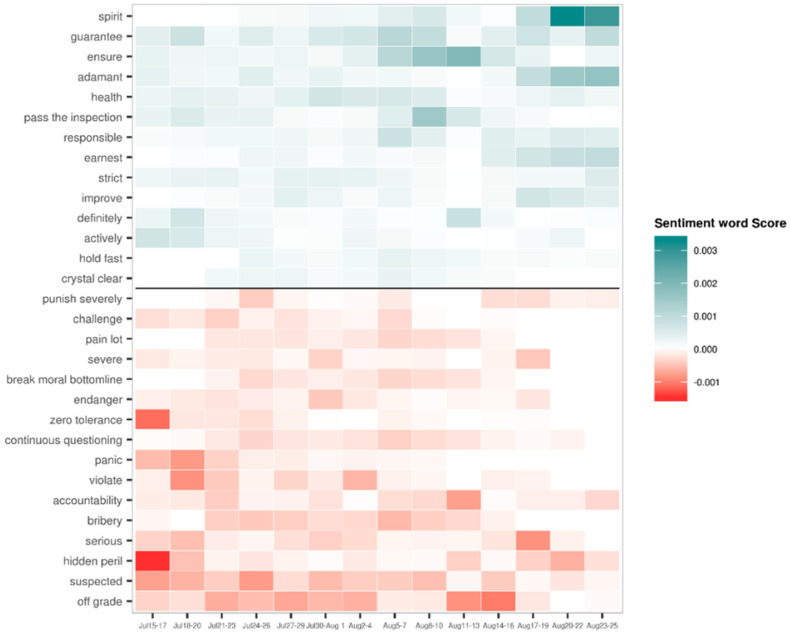
Temporal changes in the contribution of various sentiment words.

To show at what point during the observation period each word was most relevant, we analyzed how the three-day average score of each sentiment word changed over time. Figure 8 shows that negative sentiment was dominant during the initial period, with words such as “hidden peril”, “panic”, “suspected”, “zero tolerance”, and “violate” being used. During the outbreak period, both positive and negative sentiments coexisted. Once the continuation period began, the overall sentiment started to turn positive, with words such as “guarantee”, “ensure”, “health”, and “pass the inspection” emerging more frequently. The appearance of negative sentiment words such as “challenge”, “panic”, and “violate” fell rapidly to almost zero towards the end of the continuation period, particularly after 6 August. Since the dominating news topic was Final Ruling Made by the Top Leader during the ending period, the sentiment of the media was mostly positive, and the words “spirit”, “adamant”, “guarantee”, “earnest”, and “strict” appeared with high frequency.

This section may be divided by subheadings. It should provide a concise and precise description of the experimental results, their interpretations, and the experimental conclusions that can be drawn from them.

## 4. Discussion

In this study we used quantitative textual analysis to show how the media reported on the Changsheng case. While many existing studies have analyzed the news coverage of vaccination issues on a long-term scale [14,51], we focused on the response to a bursting vaccine incident over a short time span only. We began by examining the temporal trends of the incident based on news volume, and distinguished the four phases, namely, the initial period, the outbreak period, the continuation period, and the ending period (Figure 2 and Figure 3). This is in line with the life cycle of news reporting on an emergency incident, according to journalism and communication studies [52,53,54].

We further deconstructed the topics and references to the Changsheng case in a mainstream media context, and identified 17 topics falling into six categories covering Media Investigation, Response from the Top Authority, Government Action, Q&A on Vaccination, Finance Related, and Commentary. The results show that the Changsheng case, amplified by news reports, went far beyond the health sector and attracted the wide attention of the whole society [55]. This implies that the vaccine issue is not only a health issue, but a public affair that relates to politics, the economy, public security, social mentality, and so on. Further research would help determine the wider implications of the results.

Interesting findings emerged by combining the temporal and topic modeling, and illustrated a shift in focus by the media over time. For instance, news reporting focused on company investigations and business affairs during the initial period, then shifted to multiple topics during in the outbreak period, and finally narrowed their focus to topics of politics and government actions in the continuation and ending periods (Figure 3 and Figure 4). Such a shifting partly illustrates the attention of society towards the incident, and is reflective of the governmental intervention towards the case.

It is not surprising that most of the news topics during the Changsheng case had a negative sentiment propensity (Figure 5), but sentiment towards different topics also varied. Temporal sentiment shifted over the observation period, with increased negative sentiment appearing in the earlier stage of the incident (Figure 5 and Figure 8). After President Xi expressed strong resolution to stop the counterfeit and problematic production of vaccines, overall sentiment gradually turned positive, especially towards the end of the event.

In terms of policy implications, the Changsheng case was an influential public incident for which decisive governmental action and intervention were crucial, and the news reports reflected the governmental actions throughout the event. As we can see, government-related topics had the largest proportion, including confirmation of an investigation, information dissemination, immunization consultation, revaccination arrangement, and others. Such findings confirm the research of Guofeng Wang, who showed how a top-down perspective can be adopted to legitimize the ruling party and sustain social stability during a crisis [56]. The outbreak period was the window of opportunity for government intervention, and there was a quite sharp increase in news report volume during the outbreak (see Figure 2) that then dissipated quickly. This contributed to timely and adamant government action.

From a research perspective, this study shows the potential of text mining methods for vaccine related research. Compared to existing studies on the Changsheng case that have used text mining methods such as word embedding [57], conventional topic models [58], qualitative discourse analysis [56], or human-coded key phrases [59], our research employed an enhanced topic model approach with minimal subjective judgement, and combined quantified topic proportion values with temporal and sentiment features. By analyzing qualitative textual data in a quantitative way, such methods are particularly applicable to large-scale corpora when human inspection is impossible. This sheds new light on vaccine communication studies as more and more textual data emerges on the internet.

## 5. Conclusions

Using a text mining approach, this study explored the characters of news reports on a sensational vaccine case in the context of China. It showed that there were four stages in the media life cycle of the vaccine case, in which the earlier period was the opportunity window for governmental intervention. With the decisive governmental action, news reporting sentiments became increasingly positive. We presented the potential for text mining to analyze vaccine-related news text, which is also applicable to other public health issues. In terms of limitations, we only focused on official news reports and did not include other data sources such as social media, which contain more information from the user side. Furthermore, the topic model was not precise enough to incorporate more specific information at the individual level. In this case, deep learning-based natural language processing approaches would be useful for the further studies.

## Figures and Tables

**Table 1:** Topics and keywords in the news reports on the Changsheng Bio case.

Category	Label of Topics	Key Words	Proportion
Media Investigation	1. Case exposure	counterfeit, manufacturing, pharmaceutical, corporation, sales, product, Changsheng Bio.	0.034
	2. Background investigation of Changsheng Bio	Changsheng Bio, company, BioKangtai, shareholding, life sciences, 100 million-yuan, transfer, Changchun High & New Tech Industry Inc	0.036
	3. Investigation of the vaccine production chain	Changsheng Bio, sales, 100 million yuan, e10,000-yuan, sales expenditure, Changchun Changsheng, company	0.03
	4. Investigation of the problematic vaccines	DPT, issuance, off grade, potency, manufacturing, Wuhan Institute of Biological Products Co. Ltd., inspection	0.036
Response from the Top Authority	5. Commands from the top leader	work, adamant, conduct, case, investigation team, investigation, State Council	0.063
	6. Final ruling made by the top leader	regulation, meeting, pharmaceutical, problematic, work, case, safety, company	0.073
Government Action	7. Bulletin of the NMPA’s investigation	corporation, manufacturing, underway, NDA, batch, DPT, company, Changchun Bio	0.083
	8. Shangdong Provincial CDC affected	journalist, Shandong, procure, Changsheng Bio, Changchun Changsheng, DHPPi, injection	0.023
	9. Charged by the regulatory authority	company, Changsheng Bio, bulletin, Changchun Changsheng, disclose, provision, Changsheng Bio	0.081
	10. Clarification from provinces	Changsheng Bio, DPT, case, problematic, vaccination, response, off grade	0.048
	11. Explanation of the flow of the problematic vaccines	revaccination, vaccination, DPT, children, off grade, work, dose, immunization	0.063
	12. Revaccination arrangement	vaccination, rabies vaccine, Changchun Changsheng, company, revaccination, observation, CDC, national	0.108
Q&A	13. Information dissemination and consultation	vaccination, DPT, children, tetanus, kids, pertussis, off grade, batch number	0.053
Finance Related	14. Operations of Changsheng Bio affected	Changsheng Bio, company, Changchun Changsheng, manufacturing, product, freeze-dried human rabies vaccine, pharmaceutical	0.107
	15. Disturbance regarding Capital investment	project, account, Changsheng Bio, Changsheng, company, capital, journalist, investment,	0.022
	16. Delisting crisis of Changsheng Bio Stock	Changsheng Bio, fund, delisting, company, valuation, Changsheng, limit down	0.085
Commentary	17. Media commentary	problematic, case, regulation, corporation, general public, China, counterfeit, manufacturing, media	0.056

**Note:** The Key Words and Proportion columns are automatically created by the STM algorithm, and indicate the significance of the keywords within specific topics, and the semantic proportion of specific topics among the whole corpus, respectively. The Category and Label of Topics columns are annotated by the authors. Q&A: questions and answers. DPT: diphtheria, pertussis, tetanus. NMPA: National Medical Products Administration. CDC: Center for Disease Control and Prevention. Changsheng Bio (长生生物) and Changchun Changsheng (长春长生): Changchun Changsheng Biotech Co. Ltd. In Chinese.

## Supplementary Materials

The following are available online at https://www.mdpi.com/2076-393X/8/4/691/s1, 1. Table S1: Media sources of the news reports that are included in this paper. Figure S1: Semantic coherence and exclusivity of topic number 8–30.
