# Supplementary material for: Characterizing News Report of the Substandard Vaccine Case of Changchun Changsheng in China: A Text Mining Approach
Source: Vaccines (Basel). 2020 Nov 17;8(4):691. doi: 10.3390/vaccines8040691 (PMC7711875; doi:10.3390/vaccines8040691)
Supplement: Supplementary file 1 [file vaccines-08-00691-s001.pdf]

Supplementary Material

1. Data collection and cleaning procedure

To identify the articles that are relevant to the Changsheng case, we used the "Term Frequency–Inverse Document Frequency (TF-IDF)" weight of words in each document as the quantitative identifier. The TF-IDF incorporates both the frequency and relative appearance of a certain word among the whole corpus and reflect how important the word is to a document [1]. The TF-IDF weights are used in various text mining tasks such as feature extraction and document classification [2].

The specific filtering procedure in this paper is as follows:

(1) We collected 384254 news articles from 77 major news wires in China (Table 1).

We calculated the TF-IDF weight of each unique word in each document with the tidytext packages

(2) We set up a collection of keywords including Vaccine(疫苗)、Changchun Changsheng(长春长生)、Changsheng(长生)、Changsheng Bio(长生生物)、Changchun Changsheng Life Sciences Limited(长春长生生物科技有限责任公司)、Diphtheria，Tetanus and Acellular Pertussis(百白破)、freeze-dried human rabies vaccine(冻干人用狂犬病疫苗)、Diphtheria，Tetanus and Acellular Pertussis Combined Vaccine，Issue investigation (立案调查)

(3) Through manual review we included the documents in which 1) the keywords appeared at least twice uniquely, 2) the weighted mean of the keyword's TF-IDF value is larger than 0.02, and 3) the TF-IDF value of at least one the keywords ranked at the top 50 percent in all vocabulary in each document. This step filtered in3398 articles.

(4) We then removed duplicated articles by the algorithm of document distance [3]. We used the text2vec pacakges [4]. in R to remove extra documents with close distance. 2383 articles remained after this step.

Finally, we made manual confirmation of the corpus by removing unrelated articles and adding a few relevant articles. The final corpus had 2211 news articles.

Table S1. Media sources of the news reports that are included in this paper.

Source Type	Source Name	Remarks
Web portals	新浪（news.sina.com）, 搜狐(news.sohu.com), 凤凰(news.ifeng.com), 腾讯(news.qq.com), 网易(news.163.com), 人民网(news.people.com.cn), 新华网(www.xinhuanet.com), 中国新闻网(www.chinanews.com), 央广网(www.cnr.cn)	All news reports, including political news, business news, and commentaries.
Digital news media	财新网(www.caixin.com), 澎湃新闻网(www.thepaper.cn), 经济观察网(www.eeo.com.cn)	Political, economic, business, policy, and social news, and commentaries
Newspapers of the central government	People's Daily, Guangming Daily, China Youth Daily, Legal Daily, Legal Evening Paper, Farmer's Daily, China Discipline Inspection, China Women's News	Excluding international news, entertainment, sports, and advertisement.
Local newspapers	Sichuan Daily, Guangxi Daily, Jiefang Daily, Nanfang Daily, Dazhong Daily, Liaoning Daily, Tianjin Daily, Hebei Daily, Chongqing Daily, Zhejiang Daily, Shaanxi Daily, Xinhua Daily, Hubei Daily, Shanxi Daily, Henan Daily, Jilin Daily, Hunan Daily, Shenzhen News, Beijing Daily, Fujian Daily, Yunan Daily, Anhui Daily, Ningxia Daily, Xinjiang Daily, Tibet Daily, Hainan Daily, Gansu Daily, Heilongjiang Daily, Southern Metropolis Daily, Qinghai Daily, Economic Daily, Beijing News, West China City Daily, Tianfu Morning News, Xiaoxiang Morning News, Hua Shang News, Yangtse	

Evening Post, Chutian City News, Guizhou Daily, Liao Shen Evening News, Beijing Evening News, Jin Wan News, Qianjiang Evening News, Xinmin Evening News, Yangcheng Evening News, Dahe Daily, Beijing Youth Daily, Wuhan Evening News, Shenzhen Evening News, Zhengzhou Evening News, Life Daily, Inner Mongolia Daily, Jiangxi Daily, Nan Guo Morning News, Yan Zhao City News, Chongqing Evening News, City Evening News, Chun Cheng Evening News, Ningxia News, Nanguo Metropolis Daily, Lanzhou Morning News, Xi Hai Metropolis News, Guizhou Metropolis News

2. Topical number selection

In this paper, we first followed Roberts et al. to summerize semantic coherence and exclusivity of the topics with numbers ranging from 8 to 30. Figure 1 gives an overview of these two indicators among different topics, and the upper right corner indicates a better result [5]. Combined with manual review, we chose 17 as the number of topics.

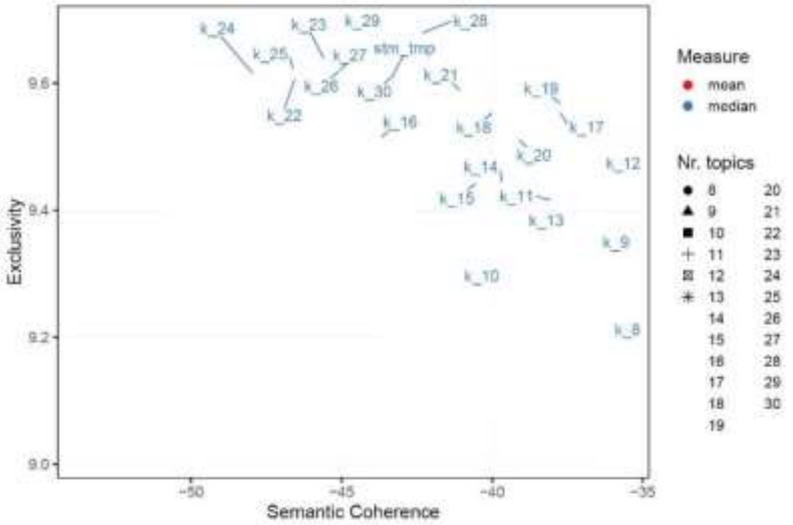

Figure S1. Semantic coherence and exclusivity of topic number 8-30.

References

1. Leskovec, J.; Rajaraman, A.; Ullman, J. *Mining of Massive Datasets*; Cambridge University Press (CUP), 2014.
2. Seki Y. Sentence Extraction by tf/idf and position weighting from Newspaper Articles. Available online: https://www.semanticscholar.org/paper/Sentence-Extraction-by-tf%2Fidf-and-Position-from-Seki/cd797cd4d225869f2eb17a5c210e04c18345132e (accessed on 17 November 2020).
3. Torres-Moreno, J.-M. *Automatic Text Summarization*; Wiley, 2014.
4. Wang DS, Qing. text2vec: Modern Text Mining Framework for R. R package version 0.5.1. 2018. Available online: https://cran.r-project.org/web/packages/text2vec/text2vec.pdf (accessed on 17 November 2020).

5. Roberts, M.E.; Stewart, B.M.; Tingley, D. stm: An R Package for Structural Topic Models. *J. Stat. Softw.* **2019**, *91*, 1–40, doi:10.18637/jss.v091.i02..
